# Phylogenetic species delimitation for crayfishes of the genus *Pacifastacus*

**DOI:** 10.7717/peerj.1915

**Published:** 2016-04-18

**Authors:** Eric R. Larson, Magalie Castelin, Bronwyn W. Williams, Julian D. Olden, Cathryn L. Abbott

**Affiliations:** 1 Department of Natural Resources and Environmental Sciences, University of Illinois at Urbana-Champaign, Urbana, Illinois, United States; 2 Pacific Biological Station, Fisheries and Oceans Canada, Nanaimo, British Columbia, Canada; 3 North Carolina Museum of Natural Sciences, Raleigh, North Carolina, United States; 4 School of Aquatic and Fishery Sciences, University of Washington, Seattle, Washington, United States

**Keywords:** Hobbsastacus, Pacifastacus fortis, Pacific Northwest, Pacifastacus leniusculus, Signal crayfish, Invasive species, Poisson Tree Processes, Cryptic species, Species delimitation

## Abstract

Molecular genetic approaches are playing an increasing role in conservation science by identifying biodiversity that may not be evident by morphology-based taxonomy and systematics. So-called cryptic species are particularly prevalent in freshwater environments, where isolation of dispersal-limited species, such as crayfishes, within dendritic river networks often gives rise to high intra- and inter-specific genetic divergence. We apply here a multi-gene molecular approach to investigate relationships among extant species of the crayfish genus *Pacifastacus*, representing the first comprehensive phylogenetic study of this taxonomic group. Importantly, *Pacifastacus* includes both the widely invasive signal crayfish *Pacifastacus leniusculus,* as well as several species of conservation concern like the Shasta crayfish *Pacifastacus fortis*. Our analysis used 83 individuals sampled across the four extant *Pacifastacus* species (omitting the extinct *Pacifastacus nigrescens*), representing the known taxonomic diversity and geographic distributions within this genus as comprehensively as possible. We reconstructed phylogenetic trees from mitochondrial (16S, COI) and nuclear genes (GAPDH), both separately and using a combined or concatenated dataset, and performed several species delimitation analyses (PTP, ABGD, GMYC) on the COI phylogeny to propose Primary Species Hypotheses (PSHs) within the genus. All phylogenies recovered the genus *Pacifastacus* as monophyletic, within which we identified a range of six to 21 PSHs; more abundant PSHs delimitations from GMYC and ABGD were always nested within PSHs delimited by the more conservative PTP method. *Pacifastacus leniusculus* included the majority of PSHs and was not monophyletic relative to the other *Pacifastacus* species considered. Several of these highly distinct *P. leniusculus* PSHs likely require urgent conservation attention. Our results identify research needs and conservation priorities for *Pacifastacus* crayfishes in western North America, and may inform better understanding and management of *P. leniusculus* in regions where it is invasive, such as Europe and Japan.

## Introduction

The conservation and management of freshwater biodiversity is dependent on recognizing this biodiversity across levels of the ecological hierarchy, from genes to ecosystems (Geist, 2011; Mace, Norris & Fitter, 2012). Molecular, and increasingly genomic, approaches have emerged over recent decades as invaluable for identifying biodiversity of conservation value that may not always be evident by traditional morphology-based taxonomy and systematics (Bickford et al., 2007). Whether identifying cryptic species (e.g., Witt, Threloff & Hebert, 2006) or evolutionary significant units (ESUs) within species (Waples, 1991), molecular approaches are now considered foundational to the field of conservation science. Although molecular approaches should not replace or marginalize traditional taxonomy and systematics, these methods do offer considerable power to clarify cases where convergent evolution (e.g., Breinholt, Porter & Crandall, 2012) or complex evolutionary histories (e.g., Niemiller, Fitzpatrick & Miller, 2008) obscure our understanding of biodiversity and consequently impair its conservation.

Freshwater crayfish are an important taxonomic group in the world’s streams, rivers, lakes, and wetlands (Crandall & Buhay, 2008). Crayfish are polytrophic, generalist consumers that can achieve high population densities and function as strong interactors in food webs (Momot, 1995). Further, crayfish are among the most imperiled freshwater taxa globally (Richman et al., 2015), while also producing some of the planet’s most harmful freshwater invasive species (Gherardi, 2007). Endemic to western North America, the crayfish genus *Pacifastacus* (Astacidae) represents a microcosm of issues in crayfish conservation and management globally (Larson & Olden, 2011). Of five modern *Pacifastacus* species, one has been declared extinct (*Pacifastacus nigrescens*), a second is listed as endangered under the United States Endangered Species Act (ESA; *Pacifastacus fortis*), two are effectively unstudied (*Pacifastacus connectens* and *Pacifastacus gambelii*), and the last is a widespread invasive species with well-documented impacts on both freshwater biodiversity and ecosystem structure and function (*Pacifastacus leniusculus*; Lodge et al., 2000; Harvey et al., 2011).

Related to these conservation and management concerns, the genus *Pacifastacus* also suffers from a complicated taxonomic history. The endangered *P. fortis* was split out of the now extinct *P. nigrescens,* first as a subspecies and subsequently as a species. Similarly, *P. connectens* was split from *P. gambelii* as a subspecies and then species. Conversely, *P. leniusculus* was initially described as three separate species that were subsequently demoted to subspecies, which now are largely ignored by most researchers and managers (reviewed in Larson & Williams, 2015). Most recently, Bouchard (1977b) recommended that *P. connectens*, *P. fortis*, and *P. gambelii* be recognized as the subgenus *Hobbsastacus* on the basis of similar mandible morphology (blade-like mandibles) relative to *P. leniusculus* (tooth-like mandibles), among other traits. Molecular genetic approaches offer great potential in resolving many of these ambiguities with respect to the taxonomy and phylogenetic relationships of *Pacifastacus* crayfishes, which in turn can direct future management and study.

We apply here a multi-gene molecular approach to investigate relationships among extant species of the genus *Pacifastacus*. In addition, we use several molecular-based species delimitation methods to gain insight into species designations independent of potentially confounding morphological characters historically used for *Pacifastacus* taxonomy. This work is motivated in part by a previous study that used a single gene to identify morphologically-cryptic diversity within crayfishes historically recognized as *P. leniusculus* (Larson et al., 2012). We significantly expand the scope of Larson et al. (2012) by increasing both taxonomic breadth and gene inclusion. Notably, this represents the first dedicated, comprehensive phylogenetic study of the crayfish genus *Pacifastacus.* These results should be of high interest both to researchers concerned with the conservation of imperiled *Pacifastacus* crayfishes in western North America (e.g., Light et al., 1995), as well as for those interested in better understanding and managing populations of *P. leniusculus* where invasive (e.g., Lodge et al., 2000).

## Materials and Methods

### Sampling and data collection

Our ingroup sampling included a total of 83 specimens representing the four extant *Pacifastacus* species. Unfortunately, we were unable to obtain tissue from the presumed extinct *Pacifastacus nigrescens* (i.e., Bouchard, 1977a). We incorporated 40 *P. leniusculus* samples (i.e., both DNA and published sequence data) from Larson et al. (2012) to: (a) represent the geographic breadth of the native range of this nominal species (*i.e., P. leniusculus sensu lato*), omitting samples from geographic areas of western North America where the species is known or suspected to have been introduced; and (b) maximize representation of the genetic variation previously observed using the 16S rDNA gene (Larson et al., 2012). We added 43 new *Pacifastacus* specimens: 11 individuals of *P. connectens* from three sites; six individuals of *P. fortis* from three sites; eight individuals of *P. gambelii* from two sites; and 18 additional individuals of *P. leniusculus sensu lato* from eight sites.

The majority (N = 31) of new specimens were collected in 2011 and 2012 by E.R. Larson and B.W. Williams, with additional individuals collected in 2012 by L. Beck of the Malheur National Wildlife Refuge (five *P. connectens* and one *P. leniusculus*) and in 2005 by M. Ellis of Spring River Ecological Sciences (all *P. fortis* specimens under US permit TE806679-3 and California permit 801319-02). New collections sought to fill in sampling gaps of Larson et al. (2012) within the range of *P. leniusculus sensu lato*, and to represent the known distributions of the *Hobbsastacus* crayfishes *P. connectens, P. fortis*, and *P. gambelii* as comprehensively as possible. For example, *P. connectens* was collected from both the endorheic (closed) Harney Basin as well as the Pacific-draining (via Columbia River) Snake River drainage, and *P. gambelii* was collected from both the endorheic Bonneville Basin and the Snake River drainage (Larson & Williams, 2015). *Pacifastacus fortis* specimens were chosen from a subset of sampled locations representing each of three genetic clusters identified through previous analysis of nine microsatellite loci (Petersen & May, 2008).

We chose five outgroup taxa to represent major lineages within the Holarctic-distributed crayfish superfamily Astacoidea based on relationships to *Pacifastacus* from recent crayfish phylogenies (Bracken-Grissom et al., 2014; Owen et al., 2015) and availability of publicly accessible data for the focal genes used in the current study. We included the Astacidae species *Astacus astacus* and *Austropotamobius torrentium* and the Cambaridae species *Cambaroides similis*, *Cambarus hamulatus* and *Orconectes virilis* (Table 1). An additional *O. virilis* specimen collected by B.W. Williams was also used, following laboratory procedures outlined below.

**Table 1:** Outgroup crayfish in phylogenetic analyses. GenBank accession numbers, with associated references, for species used as outgroups in *Pacifastacus* genus phylogenetic and species delimitation analyses across the COI, 16S, and GAPDH genes.

Species	COI	16S	GAPDH
*Astacus astacus*	AY667146^1^, GU727619*	AF235983^7^	
*Austropotamobius torrentium*	AM180946^2^	AM181346^2^	
*Cambaroides similis*	NC016925^3^	NC016925^3^	
*Cambarus hamulatus*	DQ411761^4^	DQ411739^4^	DQ411786^4^
*Orconectes virilis*	EU442729^5^, KU603541^6^	EU442672^5^, KU603569^6^	EU596269^5^, KU603606^6^

**Notes:**

1 Trontelj, Machino & Sket (2005).

2 Shubart & Huber (2006).

3 Kim et al. (2012).

4 Buhay et al. (2007).

5 Mathews et al. (2008).

6 This study.

7 Crandall, Harris & Fetzner (2000).

* Unpublished.

### DNA extraction, PCR amplification and DNA sequencing

We extracted total DNA from abdominal muscle or gill tissue using the standard protocol for the Qiagen DNeasy Blood and Tissue kit (Qiagen, Valencia, CA, USA). DNA was diluted 1:10 in ddH_2_O prior to PCR amplification. We amplified fragments of two mitochondrial genes, 16S rDNA and COI, and one nuclear locus, glyceraldehyde-3-phosphate dehydrogenase (GAPDH) using the primer pairs 16Sar-L and 16Sbr-H (Imai et al., 2004), LCOI1490 and HCO2198 (Folmer et al., 1994), and G3PCq157F and G3PCq981R (Buhay et al., 2007; Mathews et al., 2008), respectively. Amplification of COI and GAPDH products were generated by an initial denaturation step of 5 min at 95 °C followed by 35 cycles of denaturation at 95 °C for 45 s (60 s for GAPDH), annealing at 50 °C for 45 s (60 s for GAPDH) and extension at 72 °C for 2 min, with an additional final extension of 72 °C for 10 min (5 min for GAPDH). Amplification of 16S products were generated by an initial denaturation step of 3 min at 98 °C followed by 12 ‘touchdown’ cycles of denaturation at 98 °C, annealing at 50–65 °C for 30 s (with temperature decreasing by 5 °C every 3 cycles), and extension at 72 °C for 30 s, and then 30 amplification cycles with denaturation step at 98 °C for 30 s, annealing at 48 °C for 30 s, and extension at 72 °C for 30 s, and an additional final extension of 72 °C for 2 min. PCR products were purified using ExoSAP-IT (Affymetrix/USB) and sequenced bi-directionally using the above PCR primers and BigDye Terminator v 3.1 (Thermofisher). These reactions were then purified using DyeEx 2.0 Spin Kits (Qiagen) and run on a 3130xl genetic analyzer (Applied Biosystems).

Sequences were aligned using the FFT-NS-I strategy in MAFFT v. 7 (Katoh et al., 2002; Katoh & Toh, 2010; http://mafft.cbrc.jp/alignment/server/). We used BioEdit version 7.2.5 (Hall, 1999) to check for poorly aligned sites, and MEGA v 4.0 (Tamura et al., 2007) to translate protein-coding sequences (*i.e.,* COI and GAPDH) to screen for premature stop codons. We calculated number of haplotypes/genotypes, number of polymorphic sites, and number of parsimony informative sites for each gene alignment using MEGA and DnaSP v. 5.10.01 (Librado & Rozas, 2009). All new sequences were deposited in GenBank (Accession numbers: KU603429–KU603606).

### Phylogenetic analyses

Using the Bayesian Information Criterion in jModelTest 2.1.6 (Darriba et al., 2012), the suggested best-fit models of nucleotide evolution were HKI+G+I for COI and 16S genes, and TIM2ef+G for GAPDH. Three independent single-gene phylogenies were constructed using COI, 16S and GAPDH genes, excluding any redundant sequences between specimens. After checking for congruency among the tree topologies derived from the single-gene phylogenies, we conducted a fourth analysis based on concatenated sequences from a minimum of two out of the three genes analyzed. We used concatenation because this approach can outperform species tree methods when few loci are used and gene trees have low phylogenetic signal (Bayzid & Warnow, 2013; Mirarab, Bayzid & Warnow, 2014). All phylogenetic trees were built using both Maximum likelihood (ML) and Bayesian approaches (BA). First and second codon and third codon positions were used as two different partitions in COI and GAPDH analyses, whereas the noncoding 16S gene was considered a single partition. In the concatenated analysis, mutation rates were partitioned among genes, and the partitions among codon positions (first and second codon and third codon positions) were conserved for the different coding regions.

Best-scoring ML trees were estimated for each dataset using RAxML HPC2 v.8.2.4 (Stamatakis, 2006) on Teragrid v.7.2.7, implemented in the Cyber Infrastructure for phylogenetic Research (CIPRES) portal v.3.1. (Miller, Pfeiffer & Schwartz, 2010; https://www.phylo.org/). One hundred independent searches, each starting from distinct random trees, were conducted. Robustness of the nodes was assessed using nonparametric bootstrapping (Felsenstein, 1985) with 1,000 bootstrap replicates. Bayesian trees were calculated using the uncorrelated lognormal relaxed-clock model implemented in BEAST 1.8.2 with an input file generated in BEAUti version 1.8.0 (Drummond et al., 2012). When an optimal model of nucleotide evolution was not available in BEAUti 1.8.0 we selected a similar but more complex near-optimal model (Huelsenbeck & Rannala, 2004). The Yule process of speciation, which assumes a constant speciation rate among lineages, was applied as a tree prior. Each analysis ran for 100,000,000 generations with sample frequency of 1,000. The final trees were calculated based on 99,000 trees (after a burn-in of 1,000 generations) with maximum clade credibility and median node heights. Length of burn-in was determined by examination of traces in Tracer 1.5 (Rambaut & Drummond, 2009). Support for nodes was determined using posterior probabilities (PP; calculated by BEAST). In addition, a time-calibrated ultrametric tree based on the COI gene was produced using BEAST for GMYC analyses (described below). A coalescent model of constant population size was used as a tree prior and the heterogeneity of the mutation rate across lineages was set under an uncorrelated lognormal clock. The mutation rate was set to one to get branch lengths in units of substitution per site. Two independent analyses, starting from distinct coalescent trees, were run over 100,000,000 generations and sampled each 1,000 steps. After checking adequate mixing and convergence of all runs with Tracer, 10,000,000 samples were discarded as a burn-in, the two runs were pooled together and re-sampled each 20,000 steps. The maximum clade credibility tree was extracted from these results of pooled analyses using TreeAnnotator (default parameters).

### Species delimitation

Consensus support across multiple species delimitation approaches is generally preferable to reliance on a single method (Carstens et al., 2013; Fontaneto, Flot & Tang, 2015). Accordingly, we conducted species delimitation analyses using three methods: (i) the Poisson Tree Processes (PTP) method of Zhang et al. (2013); (ii) the General Mixed Yule Coalescent (GMYC, single threshold algorithm) method of Pons et al. (2006), and; (iii) the Automatic Barcode Gap Discovery (ABGD) method of Puillandre et al. (2012). We applied these methods only to the codon-partitioned COI dataset to remain consistent with a reliance on this gene fragment to identify Primary Species Hypotheses (PSHs) in animals (i.e. “DNA barcoding”; Hebert, Ratnasingham & DeWaard, 2003). Unlike ABGD that uses detection of the ‘barcode gap’ in the distribution of genetic pairwise distances, GMYC and PTP use a phylogenetic input tree from which the fit of speciation and coalescent processes are modeled to delineate PSHs (Tang et al., 2014). The ABGD method was implemented based on all available COI sequences and using the online version of the program (http://wwwabi.snv.jussieu.fr/public/abgd/) with default parameter, except that we set the relative gap width (X) to 10 to avoid the capture of smaller local gaps. The GMYC method was implemented using the time-calibrated ultrametric tree based on COI gene produced earlier using BEAST, and was run from the Exelixis Lab web server (http://species.h-its.org/gmyc/). The PTP method was implemented using the best-scoring ML tree based on COI gene produced earlier using RAxML HPC2 v.8.2.4, and was run in Python using the Environment for Tree Exploration package (Huerta-Cepas, Dopazo & Gabaldón, 2010).

## Results

### Sampling, alignment, and phylogenetic analyses

**Figure 1:**
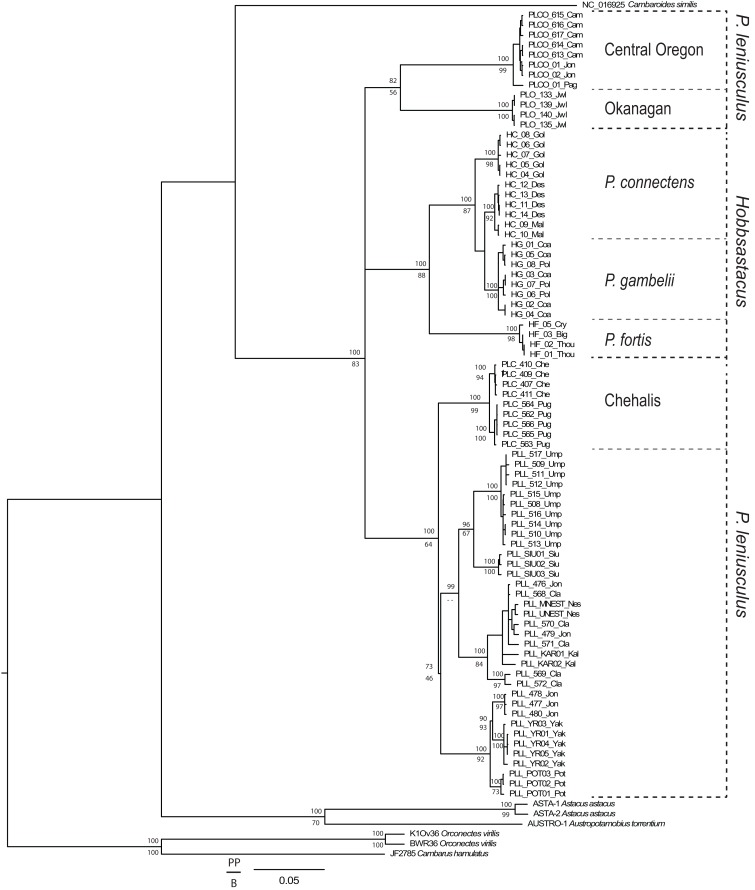
Combined gene phylogenetic tree for *Pacifastacus*. Combined gene (COI, 16S, GAPDH) Bayesian phylogenetic tree for *Pacifastacus* crayfishes, including species identities and cryptic groups (Central Oregon, Chehalis, Okanagan) proposed by Larson et al. (2012). We provide both Bayesian (posterior probability, PP; top) and maximum likelihood (bootstrap, B; bottom) support values for each node.

From the total 93 individuals used (including outgroups), we obtained COI sequences for 79 specimens (639 bp unambiguous alignment), 16S rDNA for 86 specimens (446 bp aligned, including gaps), and GAPDH for 69 specimens. We trimmed the GAPDH sequences to a final unambiguous alignment of 581 bp, representing the gene region for which we recovered fully overlapping forward and reverse sequences. For the COI gene fragment, the ingroup included 33 haplotypes displaying 149 polymorphic sites, of which 121 were parsimony informative. For the 16S gene fragment, the ingroup included 25 haplotypes displaying 42 polymorphic sites, of which 40 were parsimony informative. For GAPDH, the ingroup included 33 different genotypes displaying 20 polymorphic sites, of which 18 were parsimony informative. Phylogenetic tree topologies were congruent between Bayesian and maximum likelihood analyses. The genus *Pacifastacus* was monophyletic in the concatenated tree (Fig. 1; posterior probability value, PP = 100, bootstrap value, BS = 83) and each of the single gene analyses (Appendix S1; COI: PP = 99, BS < 50; 16S rDNA: PP = 100, BS = 65; GAPDH: PP = 76, BS = 100). Several moderately- to well-supported subclades were recovered within *Pacifastacus*, only in part reflecting morphotaxa. *Pacifastacus leniusculus sensu lato* was paraphyletic relative to a monophyletic *Hobbsastacus* subgenus, and the *Hobbsastacus* species *P. connectens* was not monophyletic in any tree.

### Species delimitation and taxonomic interpretation

**Figure 2:**
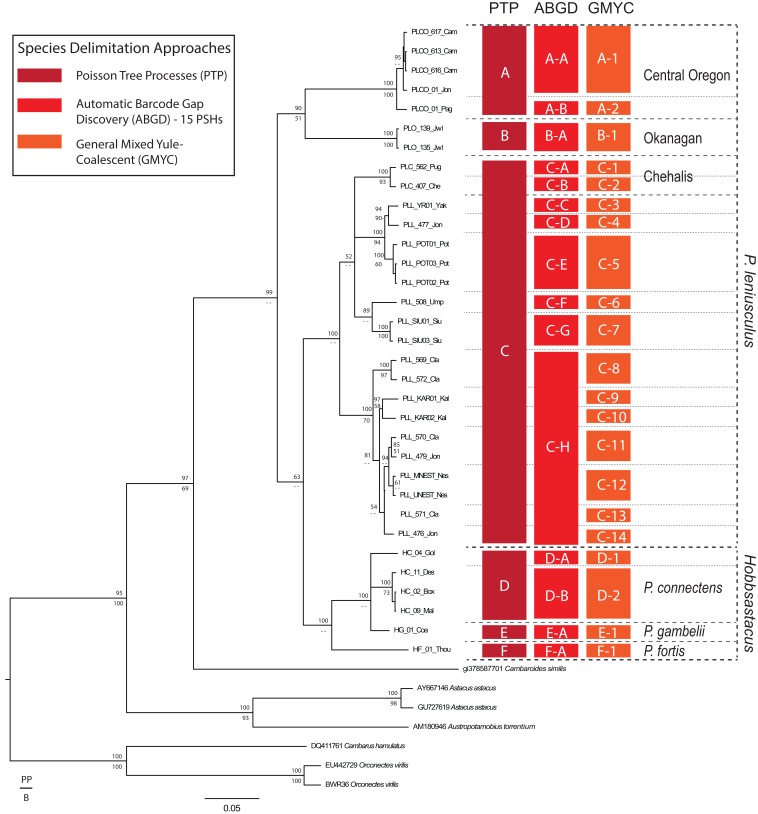
Species delimitation analyses on COI gene. Results of species delimitation analyses on the COI gene fragment using Poisson Tree Processes (PTP; Zhang et al., 2013), Automatic Barcode Gap Discovery (ABGD; Puillandre et al., 2012), and General Mixed Yule Coalescent (GMYC; Pons et al., 2006). Only the 15 Primary Species Hypothesis (PSH) partition of ABGD is reported for clarity. PSHs are labeled with letters and numbers; species of Pacifastacus crayfishes are also labeled, along with cryptic groups (Central Oregon, Chehalis, Okanagan) proposed by Larson et al. (2012). We provide both Bayesian (posterior probability, PP; top) and maximum likelihood (bootstrap, B; bottom) support values for each node on this Bayesian phylogenetic COI tree.

**Figure 3:**
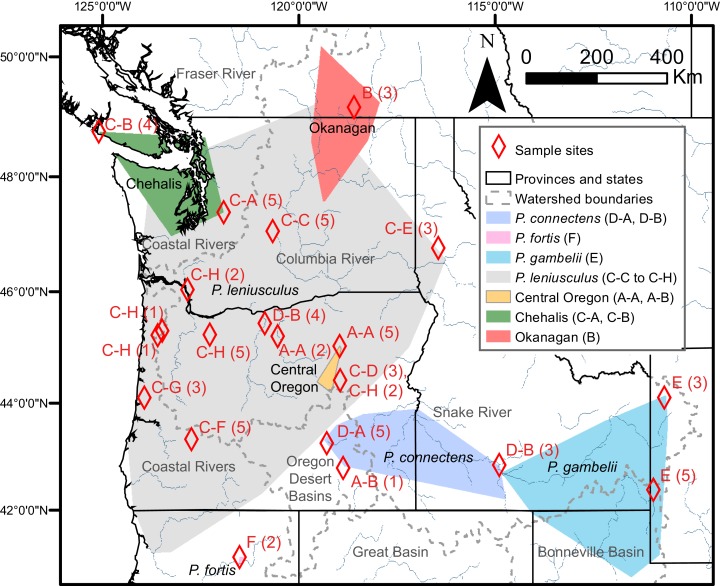
Study region with species and cryptic group ranges, as well as sample sites with Primary Species Hypotheses letters and number of samples. Map of study region, with convex hulls representing native range locations reported by Miller (1960) for the crayfish species *P. connectens*, *P. fortis*, *P. gambelii*, and *P. leniusculus* (see also Larson & Olden, 2011; Larson et al., 2012), as well as convex hulls representing all locations for three cryptic groups of *P. leniusculus* (Central Oregon, Chehalis, Okanagan) identified by Larson et al. (2012). Sampling sites used in the current study are noted by red diamonds, with the number of crayfish from each site (in parenthesis) assigned to Primary Species Hypotheses (PSHs) by species delimitation analysis on COI (Fig. 2). Codes for PSHs are based on both Poisson Tree Processes (PTP) and, in some cases, more resolved assignments from the intermediate 15 partition scenario of Automatic Barcode Gap Discovery (ABGD).

The likelihood of the null model in the PTP analysis (i.e., that all sequences belong to a single species) was found to be significantly lower than the maximum likelihood species delimitation (178 versus 226, *P* < 0.001). The PTP analysis resulted in the delimitation of six ingroup PSHs, hereafter denoted PSH-A–PSH-F (Fig. 2). *Pacifastacus leniusculus sensu lato* was delimited as three PSHs (A–C; Fig. 2). Two of the three *P. leniusculus* cryptic groups (Central Oregon, PSH-A; Okanagan, PSH-B) identified by Larson et al. (2012) delimited as PSHs, whereas the third did not (Chehalis). We do not map the three *P. leniusculus* subspecies names or morphologies to PSHs because morphological traits associated with subspecies are not conserved among phylogenetic lineages (Larson et al., 2012). All *Hobbsastacus* species delimited as separate species by PTP (*P. connectens*, PSH-D; *P. fortis*, PSH-F; *P. gambelii*, PSH-E). Sample locations with species names, cryptic group designations, and both PTP- and ABGD-delimited PSHs labels are given in Fig. 3. The GMYC method resulted in recovery of 21 PSHs within *Pacifastacus*, including 17 clusters and four entities (i.e., a single sequence representing a group). Delineations among these 21 groups did not conflict with PTP-delimited PSHs; however, additional phylogenetic species were suggested within PTP-delimited PSHs for both *P. leniusculus sensu lato* and the *Hobbsastacus* species *P. connectens* (Fig. 2). Similarly, we estimated six a priori thresholds using ABGD, resulting in six partitions with 13, 14, 14, 15, 17 and 18 PSHs. Like GMYC, ABGD results were not inconsistent with the six PSHs identified by PTP, with further splitting into additional phylogenetic species, particularly within *P. leniusculus senso lato*. Results of only the intermediate 15 PSHs partition of ABGD are given in Figs. 2 and 3.

## Discussion

Our multi-gene phylogenetic analyses, in combination with species delimitation estimation, provide evidence that *P. leniusculus sensu lato* includes morphologically cryptic diversity. Further, crayfish historically recognized as *P. leniusculus* are not monophyletic relative to the separate subgenus *Hobbsastacus* (*P. connectens, P. fortis*, and *P. gambelii*). This is similar to recent results challenging the validity of subgenera classifications in crayfishes of the family Cambaridae (e.g., Breinholt, Porter & Crandall, 2012). This cryptic diversity may ultimately merit taxonomic description as new species, albeit in consideration with other factors like sympatric coexistence or hybridization, ecological exchangeability, and morphology–in particular, tools like geometric morphometrics (e.g. Helms et al., 2015) may be useful in future applications to *Pacifastacus* crayfishes (see below). Similarly, two of our species delimitation approaches (ABGD, GMYC) identified additional PSHs within not only *P. leniusculus*, but also within *P. connectens* of the subgenus *Hobbsastacus*, relative to the more conservative PTP method. Notably, *P. connectens* was not monophyletic in any of our phylogenetic trees. Cumulatively, these results have important implications for the management and conservation of crayfishes in the Pacific Northwest of North America, as well as for regions like Europe and Japan where *P. leniusculus* is a widespread invasive species.

### Phylogenies and species delimitation

Some of the conflict between molecular-based phylogenetic approaches to classifying biodiversity and traditional morphology-based taxonomy is likely caused by a tendency for molecular phylogenetic approaches to be mutable over short time scales, owing to rapid advances in laboratory and statistical techniques, resulting in the potential misconception that they are arbitrary or indecisive. Overcoming this tension requires an appreciation that results of phylogenetic studies based on all data types, whether morphological or molecular, are not fixed, but rather are dynamic sources of data and hypotheses for subsequent inquiry, reanalysis and interpretation (Owen et al., 2015). We do not advance our analyses here as definitively overturning existing *Pacifastacus* taxonomy, or precluding future phylogenetic studies. Rather, we provide further robust support for previously reported patterns of potentially high conservation and management relevance (Larson et al., 2012), while also identifying priorities for ongoing investigation. Nevertheless, a comprehensive taxonomic revision of this genus would be useful.

In particular, several phylogenetic relationships among lineages within *Pacifastacus* remain poorly supported, in part because the fragment of the nuclear gene used in the current study (GAPDH) presented little variation within the genus. Further, these phylogenetic relationships might also be clarified by increased sampling within some of the problematic lineages in future studies. In addition, next generation sequencing and genomic approaches may advance understanding of phylogenetic relationships for *Pacifastacus* crayfishes (McCormack et al., 2013). Although the three species delimitation methods applied in this study did not achieve consensus support for number of PSHs within *Pacifastacus* (PTP = 6 PSHs; ABGD = 13–18; GMYC = 21), the results did not appear to conflict, as the divisions among PTP-defined PSHs were also recovered by GMYC and ABGD. The recovery of multiple PSHs within *Pacifastacus* combined with a failure to recover a monophyletic *P. leniusculus senso lato* relative to the *Hobbsastacus* subgenus for each gene independently supports morphologically cryptic taxa.

### Pacifastacus taxonomy

The species *P. leniusculus, Pacifastacus klamathensis*, and *Pacifastacus trowbridgii* were demoted by Miller (1960) to subspecies of *P. leniusculus* on the basis of observed intermediate morphologies proposed to represent hybridization in sympatry, a view subsequently adopted by Hobbs (1974). Unfortunately, the characters used to distinguish *P. leniusculus* subspecies are not discrete, but rather continuous gradients for several seemingly independent morphological traits. As primary examples, subspecies are distinguished by narrow (*P. l. leniusculus*) to intermediate (*P. l. trowbridgii*) to broad carapaces (*P. l. klamathensis*); long rostrums and acumens with pronounced postorbital spines (*P. l. leniusculus*) to intermediate (*P. l. trowbridgii*) to short, broad rostrums with reduced acumens and postorbital spines (*P. l. klamathensis*); and broad chelae with convex palms (*P. l. leniusculus*) to intermediate (*P. l. trowbridgii*) to narrow chelae with long, straight palms (*P. l. klamathensis*).

Miller (1960) proposed a Discriminant Function Analysis (DFA) to classify *P. leniusculus* to subspecies on a reduced set of morphological characters; Larson et al. (2012) applied this DFA to a subset of samples also considered in the current study and found that subspecies assignments were not conserved within distinct phylogenetic lineages as inferred from a fragment of the mitochondrial gene 16S rDNA. For example, *P. l. klamathensis* morphologies can be found among Central Oregon (A) and Okanagan (B) cryptic groups, the Chehalis cryptic group (C-A and C-B per ABGD), and a number of additional *P. leniusculus* PSHs reported here (e.g., C-C, C-D, and C-F per ABGD). Not all of these *P. leniusculus* lineages in disparate geographic regions can be *P. l. klamathensis.* As such, considerable work is needed to determine whether morphological attributes historically associated with *P. leniusculus* subspecies represent shared ancestral polymorphism or convergent evolution between distinct phylogenetic lineages, hybridization among them, or phenotypic responses to environmental conditions. The degree to which these distinct *P. leniusculus* lineages occur and persist in sympatry is unknown, and is likely confounded by ongoing human introductions of these crayfishes within the region (Larson et al., 2012). Even omitting the pronounced Central Oregon and Okanagan cryptic groups recognized here by the more conservative PTP analysis, the other primary lineage of *P. leniusculus senso latu* includes considerable genetic variability recovered as a diversity of PSHs by ABGD and GMYC that similarly merit future scrutiny.

*Pacifastacus connectens* was described as a subspecies of *P. gambelii* by Faxon (1914), and subsequently elevated to a species by Hobbs (1972) with little clear justification. Like the characters associated with *P. leniusculus* subspecies (above), *P. connectens* and *P. gambelii* are distinguished along gradients of several continuous morphological traits. *Pacifastacus connectens* is characterized as having a long, acute rostrum whereas that of *P. gambelii* is described as shorter and obtuse, and *P. connectens* is characterized as having long, slender chelae whereas those of *P. gambelii* are characterized as broad and robust. The degree to which these morphological characters are influenced by genotype relative to plastic responses to environment is unknown. The PTP species delimitation analysis identified *P. connectens* and *P. gambelii* as separate PSHs, whereas the ABGD and GMYC species delimitation analyses identified an additional PSH within *P. connectens*. These two *P. connectens* PSHs include a lineage sampled from several sites in the Snake River and a disjunct location in the Deschutes River (see below), and a second lineage sampled instead from the endorheic Harney Basin. Given these results in light of *Hobbsastacus* taxonomic history and potential morphological ambiguity, we propose *P. connectens* for more intensive taxonomic and phylogenetic investigation. In addition, we collected *P. connectens* from the lower Deschutes River of north Central Oregon, a major range expansion for a species never previously observed from tributaries of the lower Columbia River (Larson & Olden, 2011; Larson & Williams, 2015). We do not know whether this occurrence represents a historically overlooked component of the native range of *P. connectens*, or instead a range expansion by human introduction that is seemingly common for other species in this region (Larson & Olden, 2011; Larson et al., 2012).

### Conservation and management

The most important implication of our study is a call to better conserve and manage unique, and potentially imperiled, genetic diversity within the species historically recognized as *P. leniusculus*. For example, the Okanagan and Central Oregon cryptic groups are directly threatened by recent invasions by the virile crayfish *Orconectes virilis* and the rusty crayfish *Orconectes rusticus* (Larson & Olden, 2011). Given that the extinction and ESA listing of two other narrowly endemic *Pacifastacus* crayfishes has been attributed in part to impacts of invasive crayfish (Bouchard, 1977a; Light et al., 1995), these cryptic *P. leniusculus* are very likely at risk of population and range declines from interactions with invaders. Further work including increased taxonomic recognition of these cryptic groups will likely be necessary to motivate any conservation action. This does not necessarily require elevation to species status; for example, the Pacific Northwest region has ample experience managing ESUs of anadromous salmonids for conservation (Waples, 1991). However, ESU status is preferably based not only on genetic distinctiveness between populations, but also on ecological distinctiveness or exchangeability (Crandall, Harris & Fetzner, 2000). To date, we have no knowledge of the extent to which the genetic diversity and phylogenetic structure within *P. leniusculus* corresponds to differences in ecological function and the expression of phenotypic traits between these organisms.

Molecular approaches have enabled an increasingly resolved understanding of ways that genetic variation and its phenotypic expression can affect whole-ecosystem processes (Bailey et al., 2004; Post et al., 2008). Given that crayfish can have important but variable effects on the structure and function of freshwater ecosystems (Jackson et al., 2014), and the wide range of both genetic and phenotypic variation observed across *P. leniusculus,* it is plausible that different PSHs identified by our study are ecologically distinct. As a consequence, an investigation of this possibility has important conservation and management implications. Where introduced into California and Nevada, *P. leniusculus* has been a major invasive species and is implicated in the extinction of *P. nigrescens* and ESA listing of *P. fortis* (Bouchard, 1977a; Light et al., 1995). We do not know which *P. leniusculus* lineages or PSHs comprise these invasive populations, or how these PSHs potentially interact with each other with respect to factors like hybridization, disease or parasite transmission, or competition, which are the major mechanisms whereby native crayfish are displaced by invaders (Lodge et al., 2000). Finally, at the global scale, *P. leniusculus* is an extremely successful invasive species in Europe and Japan, with well-studied effects on other crayfish populations, freshwater communities and ecosystems, and habitat structure (Lodge et al., 2000; Usio et al., 2007; Harvey et al., 2011). Our study provides a baseline to help identify which lineages or PSHs of *P. leniusculus* are globally invasive, and to potentially identify source populations for past and ongoing introductions. This information could help guide management intervention to focus on specific introduction pathways and vectors and help slow the ongoing spread of *P. leniusculus* globally.

## Supplemental Information

10.7717/peerj.1915/supp-1 Supplemental Information 1 Individual Bayesian phylogenetic trees for the *Pacifastacus* genus on 16S, COI, and GAPDH genes.

## Additional Information and Declarations

### Field Study Permissions

The following information was supplied relating to field study approvals (i.e., approving body and any reference numbers): Pacifastacus fortis tissue was used under US permit TE806679-3 and California permit 801319-02. All other organisms were collected in compliance with state or province regulations.

### Data Deposition

The following information was supplied regarding data availability: All new sequences were deposited in GenBank (Accession numbers: KU603429–KU603606).

## References

[ref-1] Bailey JK, Schweitzer JA, Rehill BJ, Lindroth RL, Martinsen GD, Whitham TG (2004). Beavers as molecular geneticists: a genetic basis to the foraging of an ecosystem engineer. Ecology.

[ref-2] Bayzid MS, Warnow T (2013). Naïve binning improves phylogenomic analyses. Bioinformatics.

[ref-3] Bickford D, Lohman DJ, Sodhi NS, Ng PKL, Meier R, Winker K, Ingram KK, Das I (2007). Cryptic species as a window on diversity and conservation. Trends in Ecology and Evolution.

[ref-4] Bouchard RW (1977a). Distribution, systematic status and ecological notes on five poorly known species of crayfishes in western North America (Decapoda: Astacidae and Cambaridae). Freshwater Crayfish.

[ref-5] Bouchard RW (1977b). Morphology of the mandible in Holarctic crayfishes (Decapoda: Astacidae and Cambaridae): ecological and phylogenetic implications. Freshwater Crayfish.

[ref-6] Bracken-Grissom HD, Ahyong ST, Wilkinson RD, Feldmann RM, Schweitzer CE, Breinholt JW, Bendall M, Palero F, Chan T-Y, Felder DL, Robles R, Chu K-H, Tsang L-M, Kim D, Martin JW, Crandall KA (2014). The emergence of lobsters: phylogenetic relationships, morphological evolution and divergence time comparisons of an ancient group (Decapoda: Achelata, Astacidea, Glypheidea, Polychelida). Systematic Biology.

[ref-7] Breinholt JW, Porter ML, Crandall KA (2012). Testing phylogenetic hypotheses of the subgenera of the freshwater crayfish genus *Cambarus* (Decapoda: Cambaridae). PLoS ONE.

[ref-8] Buhay JE, Moni G, Mann N, Crandall KA (2007). Molecular taxonomy in the dark: evolutionary history, phylogeography, and diversity of cave crayfish in the subgenus *Aviticambarus*, genus *Cambarus*. Molecular Phylogenetics and Evolution.

[ref-9] Carstens BC, Pelletier TA, Reid NM, Satler JD (2013). How to fail at species delimitation. Molecular Ecology.

[ref-10] Crandall KA, Binida-Emonds ORP, Mace GM, Wayne RK (2000). Considering evolutionary processes in conservation biology. Trends in Ecology and Evolution.

[ref-11] Crandall KA, Harris DJ, Fetzner JW (2000). The monophyletic origin of freshwater crayfish estimated from nuclear and mitochondrial DNA sequences. Proceedings of the Royal Society B: Biological Sciences.

[ref-12] Crandall KA, Buhay JE (2008). Global diversity of crayfish (Astacidae, Cambaridae, and Parastacidae–Decapoda) in freshwater. Hydrobiologia.

[ref-13] Darriba D, Taboada GL, Doallo R, Posada D (2012). jModelTest 2: more models, new heuristics and parallel computing. Nature Methods.

[ref-14] Drummond AJ, Suchard MA, Xie D, Rambaut A (2012). Bayesian phylogenetics with BEAUti and the BEAST 1.7. Molecular Biology and Evolution.

[ref-15] Faxon W (1914). Notes on crayfishes in the U.S. National Museum and the Museum of Comparative Zoology with descriptions of new species and subspecies to which is appended a catalogue of the known species and subspecies. Memoirs of the Museum of Comparative Zoology Harvard College.

[ref-16] Felsenstein J (1985). Confidence limits on phylogenies: an approach using the bootstrap. Evolution.

[ref-17] Folmer O, Black M, Hoeh W, Lutz R, Vrijenhoek R (1994). DNA primers for amplification of mitochondrial cytochrome c oxidase subunit I from diverse metazoan invertebrates. Molecular Marine Biology and Biotechnology.

[ref-18] Fontaneto D, Flot J-F, Tang CQ (2015). Guidelines for DNA taxonomy, with a focus on meiofauna. Marine Biodiversity.

[ref-19] Geist J (2011). Integrative freshwater ecology and biodiversity conservation. Ecological Indicators.

[ref-20] Gherardi F, Gherardi F (2007). Understanding the impact of invasive crayfish. Biological Invaders in Inland Waters: Profiles, Distribution, and Threats.

[ref-21] Hall T (1999). BioEdit: a user friendly biological sequence alignment editor and analysis program for Windows 95/98 NT. Nucleic Acids Symposium Series.

[ref-22] Harvey GL, Moorhouse TP, Clifford NJ, Henshaw AJ, Johnson MF, Macdonald DW, Reid I, Rice S (2011). Evaluating the role of invasive aquatic species as drivers of fine sediment-related river management problems: the case of the signal crayfish (*Pacifastacus leniusculus*). Progress in Physical Geography.

[ref-23] Hebert PDN, Ratnasingham S, DeWaard JR (2003). Barcoding animal life: cytochrome c oxidase subunit 1 divergences among closely related species. Proceedings of the Royal Society Biological Sciences Series B.

[ref-24] Helms BS, Vaught RC, Suciu SK, Santos SR (2015). Cryptic diversity within two endemic crayfish species of the southeastern US revealed by molecular genetics and geometric morphometrics. Hydrobiologia.

[ref-25] Hobbs HH (1972). Crayfishes (Astacidae) of North and Middle America.

[ref-26] Hobbs HH (1974). A Checklist of the North and Middle American Crayfishes (Decapoda: Astacidae and Cambaridae).

[ref-27] Huelsenbeck JP, Rannala B (2004). Frequentist properties of Bayesian posterior probabilities of phylogenetic trees under simple and complex substitution models. Systematic Biology.

[ref-28] Huerta-Cepas J, Dopazo J, Gabaldón T (2010). ETE: a Python environment for tree exploration. BMC Bioinformatics.

[ref-29] Imai H, Cheng J-H, Hamasaki K, Numachi K-I (2004). Identification of four mud crab species (genus *Scylla*) using ITS-1 and 16S rDNA markers. Aquatic Living Resources.

[ref-30] Jackson MC, Jones T, Milligan M, Sheath D, Taylor J, Ellis A, England J, Grey J (2014). Niche differentiation among invasive crayfish and their impacts on ecosystem structure and functioning. Freshwater Biology.

[ref-31] Katoh K, Misawa K, Kuma KÄ, Miyata T (2002). MAFFT: a novel method for rapid multiple sequence alignment based on fast Fourier transform. Nucleic Acids Research.

[ref-32] Katoh K, Toh H (2010). Parallelization of the MAFFT multiple sequence alignment program. Bioinformatics.

[ref-33] Kim S, Park M-H, Jung J-H, Ahn D-H, Sultana T, Kim S, Park J-K, Choi H-G, Min G-S (2012). The mitochondrial genomes of *Cambaroides similis* and *Procambarus clarkii* (Decapoda: Astacidea: Cambaridae): the phylogenetic implications for Reptantia. Zoologica Scripta.

[ref-34] Larson ER, Olden JD (2011). The state of crayfish in the Pacific Northwest. Fisheries.

[ref-35] Larson ER, Abbott CL, Usio N, Azuma N, Wood KA, Herborg L-M, Olden JD (2012). The signal crayfish is not a single species: cryptic diversity and invasions in the Pacific Northwest range of *Pacifastacus leniusculus*. Freshwater Biology.

[ref-36] Larson ER, Williams BW, Kawai T, Faulkes Z, Scholtz G (2015). Historical biogeography of *Pacifastacus* crayfishes and their branchiobdellian and entocytherid ectosymbionts in western North America. Freshwater Crayfish: A Global Overview.

[ref-37] Librado P, Rozas J (2009). DnaSP v5: a software for comprehensive analysis of DNA polymorphism data. Bioinformatics.

[ref-38] Light T, Erman DC, Myrick C, Clarke J (1995). Decline of the Shasta crayfish (*Pacifastacus fortis* Faxon) of northeastern California. Conservation Biology.

[ref-39] Lodge DM, Taylor CA, Holdich DM, Skurdal J (2000). Nonindigenous crayfish threaten North American freshwater biodiversity: lessons from Europe. Fisheries.

[ref-40] Mace GM, Norris K, Fitter AH (2012). Biodiversity and ecosystem services: a multilayered relationship. Trends in Ecology and Evolution.

[ref-41] Mathews LM, Adams L, Anderson E, Basile M, Gottardi E, Bucholt MA (2008). Genetic and morphological evidence for substantial hidden biodiversity in a freshwater crayfish species complex. Molecular Phylogenetics and Evolution.

[ref-42] McCormack JE, Hird SM, Zellmer AJ, Carstens BC, Brumfield RT (2013). Applications of next-generation sequencing to phylogeography and phylogenetics. Molecular Phylogenetics and Evolution.

[ref-43] Miller GC (1960). The Taxonomy and Certain Biological Aspects of the Crayfish of Oregon and Washington.

[ref-44] Miller MA, Pfeiffer W, Schwartz T (2010). Creating the CIPRES Science Gateway for inference of large phylogenetic trees.

[ref-45] Mirarab S, Bayzid MS, Warnow T (2014). Evaluating summary methods for multi-locus species tree estimation in the presence of incomplete lineage sorting. Systematic Biology.

[ref-46] Momot WT (1995). Redefining the role of crayfish in aquatic ecosystems. Reviews in Fisheries Science.

[ref-47] Niemiller ML, Fitzpatrick BM, Miller BT (2008). Recent divergence with gene flow in Tennessee cave salamanders (Plethodontidae: *Gyrinophilus*) inferred from gene genealogies. Molecular Ecology.

[ref-48] Owen CL, Bracken-Grissom H, Stern D, Crandall KA (2015). A synthetic phylogeny of freshwater crayfish: insights for conservation. Philosophical Transactions of the Royal Society.

[ref-49] Petersen JL, May B (2008). Population and conservation genetics of Shasta crayfish (Pacifastacus fortis).

[ref-50] Pons J, Barraclough TG, Gomez-Zurita J, Cardoso A, Duran DP, Hazell S, Kamoun S, Sumlin WD, Vogler AP (2006). Sequence-based species delimitation for the DNA taxonomy of undescribed insects. Systematic Biology.

[ref-51] Post DM, Palkovacs EP, Schielke EG, Dodson SI (2008). Intraspecific variation in a predator affects community structure and cascading trophic interactions. Ecology.

[ref-52] Puillandre N, Lambert A, Brouillet S, Achaz G (2012). ABGD, Automatic barcode gap discovery for primary species delimitation. Molecular Ecology.

[ref-53] Rambaut A, Drummond A (2009). Tracer: MCMC trace analysis tool. http://tree.bio.ed.ac.uk/software/tracer/.

[ref-54] Richman NI, Böhm M, Adams SB, Alvarez F, Bergey EA, Bunn JJS, Burnham Q, Cordeiro J, Coughran J, Crandall KA, Dawkins KL, DiStefano RJ, Doran NE, Edsman L, Eversole AG, Füreder L, Furse JM, Gherardi F, Hamr P, Holdich DM, Horwitz P, Johnston K, Jones CM, Jones JPG, Jones RL, Jones TG, Kawai T, Lawler S, López-Mejía M, Miller RM, Pedraza-Lara C, Reynolds JD, Richardson AMM, Schultz MB, Schuster GA, Sibley PJ, Souty-Grosset C, Taylor CA, Thoma RF, Walls J, Walsh TS, Collen B (2015). Multiple drivers of decline in the global status of freshwater crayfish (Decapoda: Astacidea). Philosophical Transactions of the Royal Society B.

[ref-55] Shubart CD, Huber MGJ (2006). Genetic comparisons of German populations of the stone crayfish, *Austropotamobius torrentium* (Crustacea: Astacidae). Bulletin Francais de la Pêche et de la Pisciculture.

[ref-56] Stamatakis A (2006). RAxML-VI-HPC: maximum likelihood-based phylogenetic analyses with thousands of taxa and mixed models. Bioinformatics.

[ref-57] Tamura K, Dudley J, Nei M, Kumar S (2007). MEGA4: Molecular Evolutionary Genetics Analysis (MEGA) software version 4.0. Molecular Biology and Evolution.

[ref-58] Tang CQ, Humpreys AM, Fontaneto D, Barraclough TG (2014). Effects of phylogenetic reconstruction method on the robustness of species delimitation using single-locus data. Methods in Ecology and Evolution.

[ref-59] Trontelj P, Machino Y, Sket B (2005). Phylogenetic and phylogeographic relationships in the crayfish genus *Austropotamobius* inferred from mitochondrial COI gene sequences. Molecular Phylogenetics and Evolution.

[ref-60] Usio N, Nakata K, Kawai T, Kitano S (2007). Distribution and control status of the invasive signal crayfish (*Pacifastacus leniusculus*) in Japan. Japanese Journal of Limnology.

[ref-61] Waples RS (1991). Pacific salmon, *Oncorhynchus* spp., and the definition of “species” under the Endangered Species Act. Marine Fisheries Review.

[ref-62] Witt JD, Threloff DL, Hebert PDN (2006). DNA barcoding reveals extraordinary cryptic diversity in an amphipod genus: implications for desert spring conservation. Molecular Ecology.

[ref-63] Zhang J, Kapli P, Pavlidis P, Stamatakis A (2013). A general species delimitation method with applications to phylogenetic placements. Bioinformatics.

