# Supplementary figures and images for: Phylogenetic species delimitation for crayfishes of the genus Pacifastacus
Source: PeerJ. 2016 Apr 18;4:e1915. doi: 10.7717/peerj.1915 (PMC4841241; doi:10.7717/peerj.1915)

## col

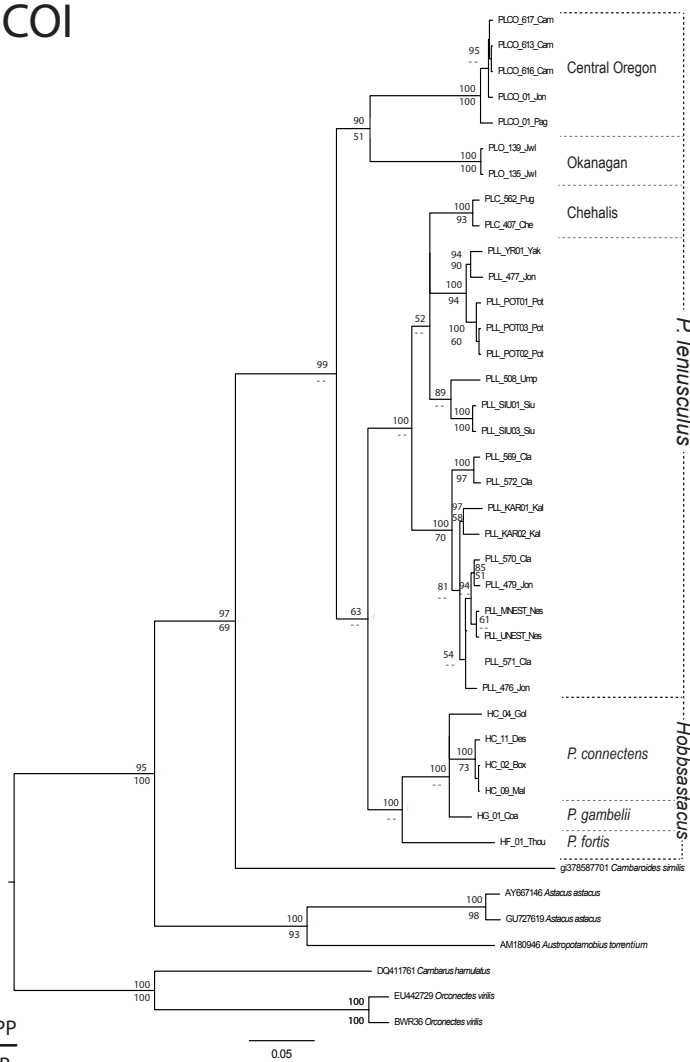

# 16S

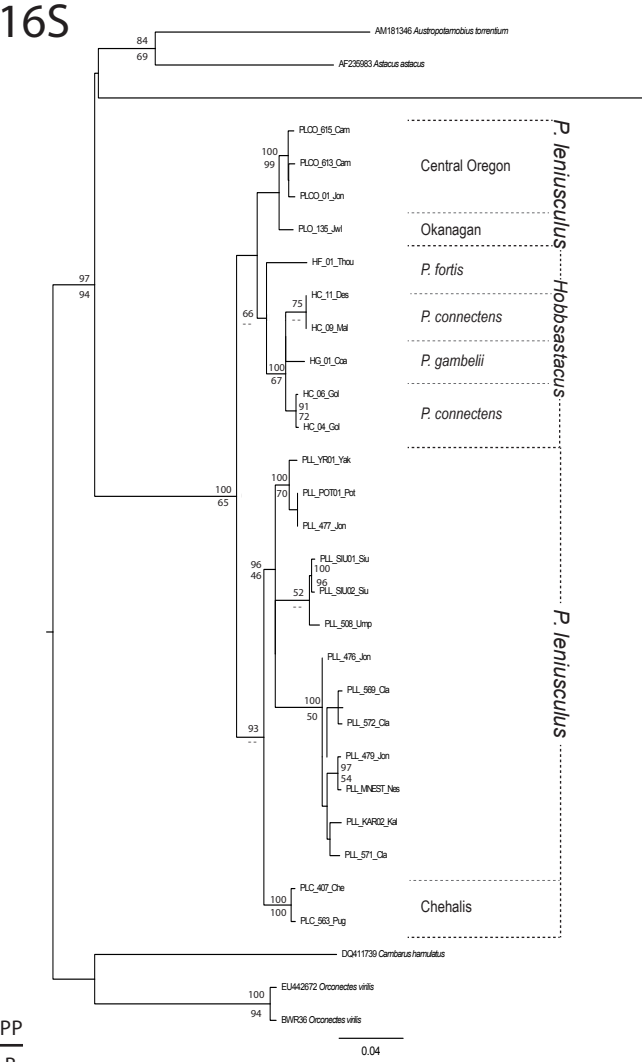

# GAPDH

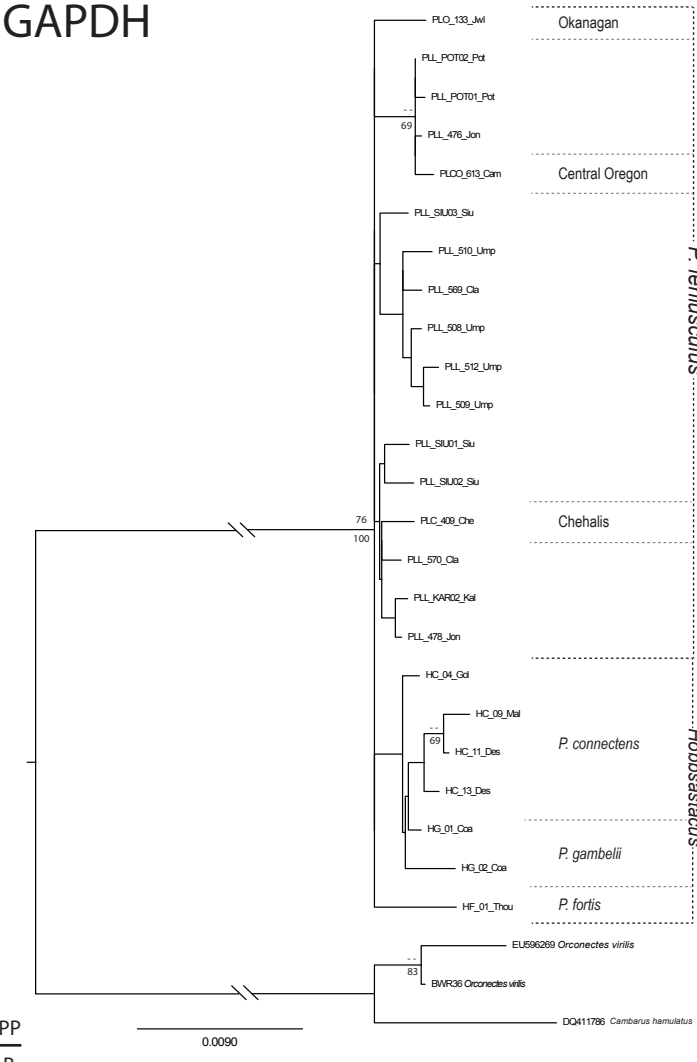

Supplement: Supplemental Information 1 [file peerj-04-1915-s001.pdf]
